# Virtual Reality Not for “Being Someone” but for “Being in Someone Else's Shoes”: Avoiding Misconceptions in Empathy Enhancement

**DOI:** 10.3389/fpsyg.2021.741516

**Published:** 2021-08-24

**Authors:** Francisco Lara, Jon Rueda

**Affiliations:** Department of Philosophy, University of Granada, Granada, Spain

**Keywords:** empathy, empathy enhancement, moral enhancement, sympathy, virtual reality ethics

## Introduction

Ramirez et al. (2021) have recently claimed that using Virtual Reality (VR) as an educational nudge to promote empathy is unethical. These authors argue that the influence exerted on the participant through virtual simulation is based on the deception of making them believe that they are someone else when this is impossible (p. 4–7). This makes the use of VR for empathy enhancement a manipulative strategy in itself.

Following Goldie (2011, but see also Goldie, 1999, 2000), Ramirez et al. argue that adopting the other's perspective is very difficult or even impossible. In most cases, in order to adopt the other's perspective, one must govern his or her imaginative process with a “characterisation” of the other, based on past experiences and (sometimes unconscious) aspects of his or her character or emotions, to which the observer does not have access. They reinforce this argument with some insights from semantic variance and intersectional approaches to personal identity. In a nutshell, they claim that the promise of being “someone else” in VR is doomed to fail. Consequently, empathy-enhancing simulations are deceptive in nature and, therefore, they constitute an ethically undesirable educational tool. Alternatively, the authors suggest that it would be more justified to use VR to enhance sympathy—understanding “sympathy” as the “process of *feeling for* another” which leads to expressing an “attitude of care or of concern with respect to their target” (p. 13, italics in the original source).

In this article, we show that Ramirez et al.'s ethical rejection of empathy enhancement through VR is based on confusion. First, we show that this misunderstanding stems from the conception of empathy-enhancing simulations solely as failed attempts at “being someone else,” along with ignoring the crucial difference between the psychological perspective-taking processes of imagine-other and imagine-self. Then, having overcome that misconception, we argue that the ethical misgivings about the use of VR to promote empathy should disappear and that these projects have greater potential for behavioural change than purely sympathy-focused interventions.

## Empathy: Whose Perspective-Taking?

Ramirez et al. (2021, p. 7) recognise that empathy understood as perspective-taking—or what Darwall (1998) called “projective empathy” or “simulation”—essentially admits two modes. According to Goldie (2011, p. 302), on the one hand, one can aim to imagine the perspective of *other* subjects, thus assuming *their* thoughts, feelings, decisions and diverse aspects of their psychology. On the other hand, one can share someone's perspective by imagining *oneself* in the other person's shoes. In other words, in this second mode you would seek to get an idea of which thoughts, feelings, decisions or other psychological traits you would come up with if *you* were in the other person's circumstances. These two forms of perspective-taking (imagine-other and imagine-self perspectives) have often been confused or equated with each other despite empirical evidence of their differences (Stotland, 1969; Batson et al., 1997a).

Although Ramirez et al. do not confuse this distinction, we believe that their argument does not give enough relevance to the differences between them. An example of this is that Ramirez et al. (2021, p. 7) refer to a text by Goldie (2011, p. 309) to defend the infeasibility of any perspective-taking proposal in VR when, with this text, and with his entire article, Goldie intended to criticise only “empathetic perspective-shifting” or imagine-other perspective-taking, and not “in-her-shoes perspective-shifting” or imagine-self perspective-taking (Goldie, 2011, p. 302).

These two forms of perspective-taking are not merely conceptually different. Some empirical studies have shown that both processes require different skills (e.g., imagine-other requires greater mental flexibility and emotional regulation to put one's perspective in abeyance) and have different effects (Batson et al., 1997b, 2003; Goldie, 1999, 2000, 2011; Decety and Chaminade, 2003; Decety and Sommerville, 2003; Decety, 2006, 2007; Decety and Grézes, 2006; Decety and Hodges, 2006; Decety and Jackson, 2006; Goldman, 2006, 2011; Iacoboni, 2008). Moreover, they activate different neurological mechanisms (Ruby and Decety, 2001, 2004; Jackson et al., 2006a). By default, when we try to imagine the perspective of others we tend to do so with an in-her-shoes simulation (Keysar et al., 2003; Royzman et al., 2003; Goldman, 2006; Jackson et al., 2006b). Therefore, keeping this distinction in mind is very important for the discussion on the ethical aspects of enhancing empathy through VR.

## Discussion

We agree with Ramirez et al. that using VR to simulate being a specific other person is very difficult because of the real disparity between the two individuals involved and the observer's impossible access to prior experiences and sensations important for really understanding what it means to be that other person. In that sense, presenting VR as being able to achieve what it cannot really do—allowing us to be “someone else”—could be misleading. Still, we also believe that expectations can be much better if VR is used not to pretend to be someone else but to represent other people's perspectives without pretending to know their personal identity entirely, that is, to put yourself in their shoes but still be yourself.

However, this kind of “imagine-self” perspective-taking also has its problems. The most significant has to do with our intrinsic egocentric bias. When we try to guess how the other thinks, feels or desires we have a tendency to assume a greater similarity between them and us than actually exists (Keysar et al., 2003), leading to prediction errors about others' behaviour and mental state (Dunning et al., 1990; Goldman, 2006; Coplan, 2011, p. 10–11).

Having said that, we believe that this type of VR simulation can be feasible and educationally beneficial. Some strategies could be applied in this respect. On the one hand, complementary measures of transparency can be taken so as not to be accused of being deceptive. For instance, before the virtual experience, participants could be told that the embodiment in avatars of other social groups does not exactly imply being like a person from those collectives. Such warnings would help us to circumvent the objection of deception and would also serve to preserve the possible misgivings of the members of the social groups represented.

On the other hand, the key would be to use VR in a restricted way, especially attempting to help the user to more easily imagine what it is like, not to be someone in particular, but to experience certain typical, simple and very generalisable situations. Thanks to the use of virtual avatars that simulate the experience of having different bodies, it can be easier to imagine adopting the perspective of another—even an animal (Ahn et al., 2016) or a superhero (Rosenberg et al., 2013), while still being able to differentiate that embodiment from our human identity. It would be a matter of mainly embodying the virtual representation of the specific out-group trait that is intended to increase empathy.

It would also help to underline that this empathy-enhancing use of VR is for educational^1^ or training purposes, and that it voluntarily cultivates the ability to put oneself in the other's shoes (Persson and Savulescu, 2018, pp. 186, 190; Read, 2019). Moreover, through virtual embodiment, what is achieved is only to make these experiences “more experientially vivid” than simply imagining the other's point of view with traditional perspective-taking methods (Gehlbach et al., 2015).

From this concrete application of VR, the predictive difficulties that could be objected to in-her-shoes empathy (due to its egocentric bias) could be avoided. In part, because if the simulation aims to acquire better knowledge of only a certain (simple and universal) aspect of the other person's perspective, and not all the details of it, the fact that the observer cannot make accurate predictions about the other person's thoughts and behaviour as a whole would lose its relevance. Moreover, if necessary, unlike in a traditional (non-technologically mediated) simulation exercise, the designer can provide the avatar software with the data that the user does not have about the target character, or introduce operational strategies to block or counteract the user's egocentric bias.

Furthermore, we believe that this restricted in-her-shoes virtual embodiment could be more effective than the alternative proposal by Ramirez et al. to nudge users' sensitivity to the suffering and injustices of others. These authors advocate VR simulations that seek sympathy rather than empathy. In their own words, “(s)ubjects of those simulations are invited to see the simulations as something they are witnessing and hence don't involve problematic perspective-taking” (p. 13). Users would thus be no more than “engaged witnesses” or “sympathetic bystanders.” Ramirez et al. call for simulations to be constructed in such a way that they “provide the subject with their own point-of-view as opposed to the point-of-view of a different person.” (p. 13). This undermines the possibility of virtual embodiment because it entails taking the first-person perspective of another subject. They support this by stressing that, thanks to this substantive distancing of the viewpoints involved, sympathy will not be about the empathy-related “mirroring the feelings of another,” but rather about expressing “an attitude of care or concern with respect to their target” (p. 13).

Indeed, we agree that there should be a differentiation between the viewpoints of the observer and the target, but that this is primarily due to cognitive factors. It will be much more effective in terms of awareness-raising if the differentiation of viewpoints does not entail the detachment related to the attitude of the mere spectator (see Ventura et al., 2020). In contrast to this detachment, it is worth noting that a distinctive feature of the in-her-shoes virtual embodiment is its high likelihood of giving rise to an “empathic concern,” that is, greater compassion and interest for those suffering from negative experiences and a greater willingness to alleviate it (Davis, 1980). Therefore, it turns out that, even if one's own perspective is implanted in that of others with an essentially cognitive purpose, the result can be a feeling on the behalf of the other, in many cases because of the selfish interest of avoiding the anguish produced by this experience (Batson et al., 1997a).

Thus, unlike sympathy enhancement, our proposal of empathy enhancement through virtual embodiment seeks to improve the understanding of certain emotions of others to reinforce motivation, namely, provoking greater concern for the target that translates into effective changes in behaviour (Rueda and Lara, 2020). Let us not forget that I would be imagining what it would be like for *me* to be in the painful situation of the other, as opposed to the proposal of Ramirez et al. in which the pain would always be presented as that of the *other*. Elsewhere we have reviewed an increasing body of evidence of how Virtual Reality Embodied Perspective-Taking (VREPT) experiences have promising behavioural changes in the real world (Rueda and Lara, 2020, p. 7–11). Virtual embodiment refers to the experience of inhabiting another virtual subject by the process of body transfer into a digital avatar. Virtual embodiment can be used to adopt the perspective of different out-group people in order to increase the empathy to concrete social targets through embodying their digital representations. Some experiments have placed participants in the bodies of virtual avatars with other skin tones (Groom et al., 2009; Peck et al., 2013; Banakou et al., 2016; Hasler et al., 2017), different genders (Seinfeld et al., 2018), ages (Oh et al., 2016; Hamilton-Giachritsis et al., 2018), members of disabled groups (Ahn et al., 2013; Chowdhury et al., 2019), people in situations of extreme social exclusion (Herrera et al., 2018), and even other species (Ahn et al., 2016). Seinfeld et al.'s (2018) experiment in which some male batterers who adopted the perspective of abused women with female avatars increased their ability to recognise emotions in women's faces may serve as a prominent example. Presumably, VREPT is more likely to lead to greater awareness of the problem than if VR were only intended as a simulation in which the user does not leave the role of bystander.

Summarising, Ramirez et al.'s disbelief regarding ethical appraisal of empathy-enhancing simulations in VR is surmountable. Although VR does not allow us to be exactly “someone else,” it permits us to inhabit other virtual avatars through the embodiment in the representations of other social identities. Walking in their virtual shoes can be a significant step towards the ethical permissibility of empathy enhancement in VR.

## Author Contributions

FL and JR contributed to the bibliography exploration, content development, and writing of the manuscript. Both authors listed have made a substantial, direct and intellectual contribution to the work, and approved it for publication.

## Conflict of Interest

The authors declare that the research was conducted in the absence of any commercial or financial relationships that could be construed as a potential conflict of interest.

## Publisher's Note

All claims expressed in this article are solely those of the authors and do not necessarily represent those of their affiliated organizations, or those of the publisher, the editors and the reviewers. Any product that may be evaluated in this article, or claim that may be made by its manufacturer, is not guaranteed or endorsed by the publisher.

## Footnotes

^1^For instance, the research of Ingram et al. (2019) with middle school students showed that virtual reality interventions decreased traditional bullying perpetrations mediated by an increase in empathy.
